# Effective Population Size, Genetic Variation, and Their Relevance for Conservation: The Bighorn Sheep in Tiburon Island and Comparisons with Managed Artiodactyls

**DOI:** 10.1371/journal.pone.0078120

**Published:** 2013-10-11

**Authors:** Jaime Gasca-Pineda, Ivonne Cassaigne, Rogelio A. Alonso, Luis E. Eguiarte

**Affiliations:** 1 Departamento de Ecología Evolutiva, Instituto de Ecología, Universidad Nacional Autónoma de México, México City, México; 2 Facultad de Medicina Veterinaria and Zootecnia, Universidad Nacional Autónoma de México, México City, México; CSIRO, Australia

## Abstract

The amount of genetic diversity in a finite biological population mostly depends on the interactions among evolutionary forces and the effective population size (*N*_*e*_) as well as the time since population establishment. Because the *N*_*e*_ estimation helps to explore population demographic history, and allows one to predict the behavior of genetic diversity through time, *N*_*e*_ is a key parameter for the genetic management of small and isolated populations. Here, we explored an *N*_*e*_-based approach using a bighorn sheep population on Tiburon Island, Mexico (TI) as a model. We estimated the current (*N*_*crnt*_) and ancestral stable (*N*_*stbl*_) inbreeding effective population sizes as well as summary statistics to assess genetic diversity and the demographic scenarios that could explain such diversity. Then, we evaluated the feasibility of using TI as a source population for reintroduction programs. We also included data from other bighorn sheep and artiodactyl populations in the analysis to compare their inbreeding effective size estimates. The TI population showed high levels of genetic diversity with respect to other managed populations. However, our analysis suggested that TI has been under a genetic bottleneck, indicating that using individuals from this population as the only source for reintroduction could lead to a severe genetic diversity reduction. Analyses of the published data did not show a strict correlation between *H*_*E*_ and *N*_*crnt*_ estimates. Moreover, we detected that ancient anthropogenic and climatic pressures affected all studied populations. We conclude that the estimation of *N*_*crnt*_ and *N*_*stbl*_ are informative genetic diversity estimators and should be used in addition to summary statistics for conservation and population management planning.

## Introduction

It has been recognized that management strategies should consider non-genetic factors (e.g., demography) as well as genetic factors (e.g., genetic drift and inbreeding depression) [1,2]. Although it has been argued that non-genetic factors could be more relevant for conservation [3,4], it is accepted that minimizing the loss of genetic variation is a major goal for the management of small populations [1,5]. Actually, the amount of genetic diversity is considered a parameter for biological conservation [5–7]. In this context, a primary factor responsible for the rate of loss of genetic diversity as well as the rate of increase of inbreeding and genetic drift in a biological population is the effective population size (*N*_*e*_) [6,8–10]. Therefore, the estimation of *N*_*e*_ has recently become a commonly used parameter in population genetics studies of endangered species [10–14]. Recent methods approximate past changes in *N*_*e*_, allowing the inference of the population history by estimating changes in historical *N*_*e*_ [15–17]. This makes it feasible to distinguish between historical and recent human-influenced levels of genetic diversity [18,19]. Moreover, the estimation of *N*_*e*_ allows one to predict the behavior of genetic diversity under different demographic scenarios using computational simulations [15,18].

The effective population size (*N*_*e*_) is defined as the size of a simple Wright-Fisher population that would have the same increase in homozygosity and the same random drift in allele frequencies as in the actual population considered [8,20–22]. Thus, the definition and estimation of *N*_*e*_ will depend on the feature or genetic property of interest, such as the loss of genetic diversity due to genetic drift or inbreeding [22]. In conservation genetics studies, the most widely used definitions [13,15] are the variance effective population size *N*_*eV*_, which measures the variance of change in gene frequency through time due to genetic drift, and the inbreeding effective population size *N*_*eI*_, which is defined in terms of the probability that two individuals have the same parent (i.e., identical by descent) [14,22]. In other words, *N*_*eI*_ measures the loss of expected heterozygosity due to relatedness [12]. Based on these definitions, several methods for estimating *N*_*e*_ have been proposed [8,11–15,22,23]. Nonetheless, it is important to bear in mind that certain methods have particular assumptions or data requirements that could be applied to specific cases [8,12,15–17].

### Tiburon Island Bighorn Sheep Population

**Figure 1:**
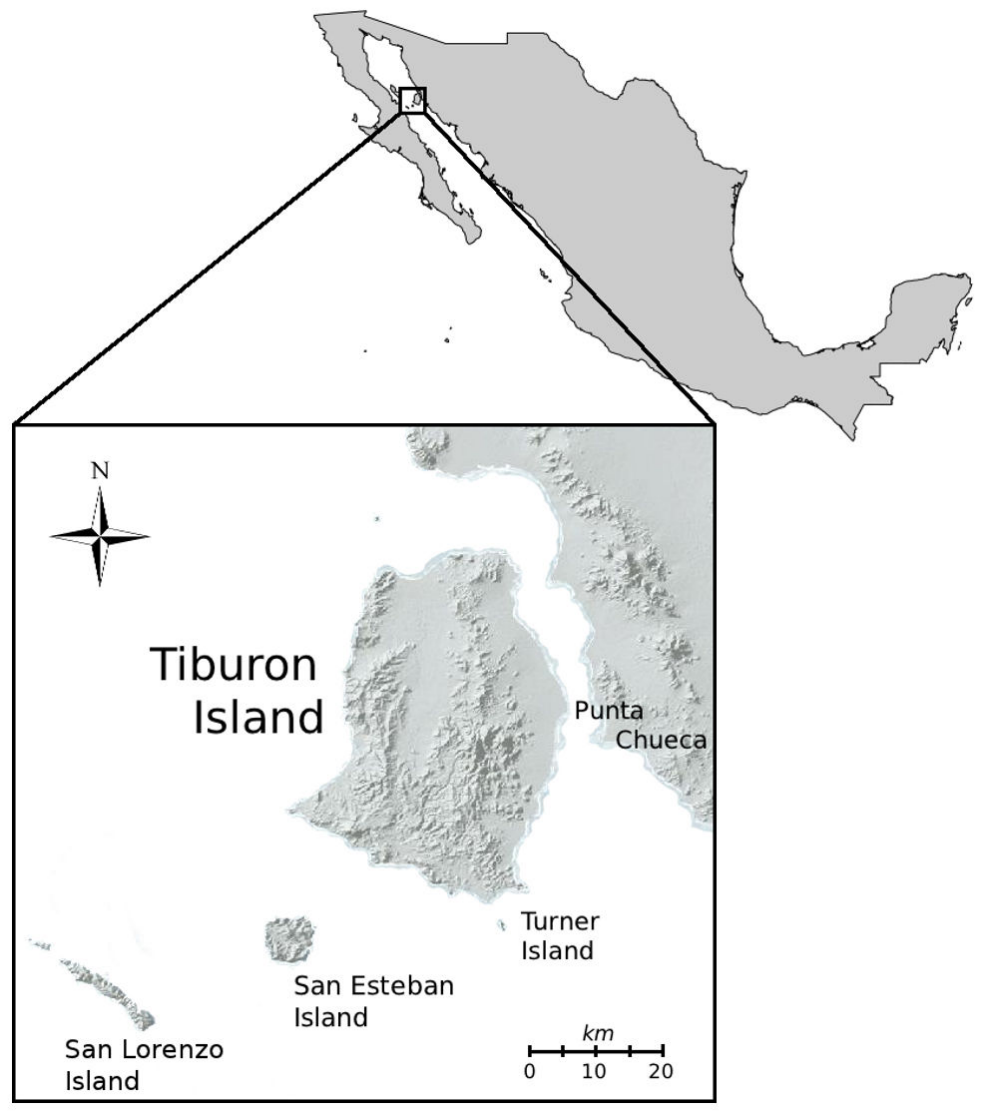
Map showing the localization of Tiburon Island on the Gulf of California, Mexico.

The bighorn sheep (*Ovis canadensis*) population on Tiburon Island (TI) was founded in 1975 by a single introduction event of 16 individuals (14 ewes and 2 rams) from Punta Chueca Sonora, Mexico [24] (Figure 1). After this event, the population remained isolated [25–27], and in less than 20 years, the population increased to ~700 individuals [25,27]. This suggests that the TI population has not (at least not yet) shown issues associated with demographic trends, even it has been proposed that the island is near its carrying capacity [25].

Previous studies underlined the importance of retaining genetic diversity in bighorn sheep [28–31], especially when new populations are founded [28,29]. In general, it has been assumed that the number of founders determines the magnitude of the genetic diversity in captive populations [32–34], although there are some exceptions [35]. This raises relevant questions. Did the founders used represent an "adequate" sampling of genetic diversity (i.e., no original genetic bottleneck)? What can the effects on genetic diversity be for using individuals from TI as the only source for founding new populations?

Hedrick et al. [36] conducted the first study that explored the genetic diversity and its impact on *N*_*e*_ in TI. They analyzed a limited number of samples (14 individuals, 10 microsatellite loci and 1 MHC locus) and found a lower effective number of founders than the actual number of individuals introduced (14 founders vs. 16 introduced). Recently, another study was published estimating the genetic diversity of the TI population [37], reporting *H*_*E*_, *H*_*O*_ and *F'* statistics.

In this study, we explored an inbreeding effective population-size-based approach to assess the genetic diversity of the TI population, and the demographic scenarios that could explain such diversity, including its foundation and historical events. In addition, we estimated the possible outcome in terms of *N*_*eI*_ and *H*_*E*_ of using the TI population as a source of individuals for founding new populations. Finally, to compare our approach, we re-analyzed previously published microsatellite allelic data from seven *O. canadensis* populations and three other artiodactyl species.

## Methods

### Ethics Statement

The work met the Mexican legal requirements about animal welfare and field work, and was supervised and approved by Dirección General de Vida Silvestre (DGVS), Secretaría de Medio Ambiente y Recursos Naturales (SEMARNAT). The hair sampling was performed in accordance with the Mexican official standard NOM-062-ZOO-1999 (dealing with specific techniques for production, care, and use of laboratory animals). Because hair sampling is a non-invasive technique, it was not necessary to ask for the Institutional SICUAE (Subcomité Institucional para el Cuidado y Uso de Animales de Experimentación) approval.

The sampling in Tiburon Island was conducted under the Seri Government permission, as well as with permission of the owners of "Rincón de la Madera" and "La Mesa" UMAs (Unidad de Manejo para la Conservación de la Vida Silvestre; Management Unit for Wildlife Conservation). The special permit for sample collection was issued under Mexican law by DGVS and SEMARNAT.

### Population sampling and DNA extraction

Tiburon Island is located in the Gulf of California near the coast of Sonora, Mexico (Figure 1). The island has an abundant, suitable habitat for bighorn sheep (*Ovis canadensis*), including mountain ranges and typical continental vegetation dominated by deciduous shrubs and succulent cacti [25,38]. Hair samples were collected from 63 animals captured along the different mountain ranges in Tiburon Island in 2007 (Figure 1). At the time of capture (October), ewes and rams were split and many different herds were found on the island; thus, the sampling was intended to include as many herds as possible. Hair samples were stored in paper envelopes at -80°C until used. DNA was extracted from 15–25 hairs per individual using a standard Chelex (Chelex 100, Bio-Rad) protocol [39,40].

### Microsatellite genotyping and mitochondrial sequencing

To evaluate the nuclear genetic diversity, we used a set of 12 microsatellite loci isolated from *Ovis aries* or *Bos taurus* (Table S1). These markers had been reported as polymorphic and non-linked. Additionally, some of them had already been used in other genetic diversity studies on bighorn sheep populations [28,36,41]. The Polymerase Chain Reaction (PCR) conditions and primer sequences are reported in Table S1 and Protocols S1. In order to reduce possible genotyping errors, 30% of the sample was re-amplified at random for each locus. To detect possible bias due to stutter, allele dropout, and/or null alleles, we used the statistical tests implemented in Micro-checker version 2.2.3 [42]. In addition, the mitochondrial control region was amplified using the primers previously reported by Boyce et al. [43]. However, three internal primers were designed to obtain shorter fragments (primer sequences and conditions are reported in Protocols S1). Fragment assembly and nucleotide quality assessment were carried out with Consed version 1.9 [44,45]. The sequence alignment was done with ClustalX version 2.1 [46].

### Population genetics analysis

To estimate the genetic diversity of the microsatellite data of the TI population, we calculated the expected heterozygosity (*H*_*E*_), observed heterozygosity (*H*_*O*_), and number of alleles (*n*_*a*_), as well as its respective standard deviation (S.D.), using Arlequin version 3.5 [47]. Tests for departures from Hardy-Weinberg expectations through the values of *F*_*IS*_ statistic (fixation index, [48,49]) were carried out using the Genepop software, version 4.1 [50]; the significance of the estimated *F*_*IS*_ value was calculated using the Fisher exact test with 10,000 dememorisation steps, 1,000 batches, and 5,000 iterations per batch. We also used Genepop to test for linkage disequilibrium for each pair of loci using the Fisher exact test with the same settings reported above. To detect within-island genetic structures, we used STRUCTURE version 2.3 [51] with 500,000 MCMC iterations followed by a burning period of 250,000 steps. As the TI population was founded using individuals from the same location, the admixture model was implemented [52]. The number of clusters (k) varied from 1 to 10 with 30 iterations for each one. Then, to obtain the most likely value of *k*, the Evanno et al. [53] method was applied using Structure Harvester [54]. However, this method excludes the two extreme values of *k* (in this case, 1 and 10), so all LnP(D) values were plotted against *k* to visually inspect its behavior and choose the *k* value with higher likelihood and lower variance. The genetic diversity analysis of the mitochondrial sequences were carried out estimating the number of haplotypes, nucleotide diversity (π), and haplotype diversity using DnaSP version 5.10 [55].

### Population size changes and inbreeding effective size (N_eI_) estimation

We used the method proposed by Beaumont [56] and Storz and Beaumont [57], which considers the inbreeding effective size definition (*N*_*eI*_) [12]. This method explores hypotheses about the historical signal of demographic expansion or contraction in a closed population through coalescent simulations. Then, each hypothesis is evaluated and the parameters values are estimated via Bayesian inference. We chose this method because it does not require life history data and it only uses a single temporal sampling. Moreover, it allows variation on parameters from one locus to the next one (as different mutation rates), so it is suitable for multilocus microsatellite analyses. In addition, it has been used to estimate population size changes of several species, including lemurs [58] and martens [59]. Finally, this method has demonstrated its robustness for detecting past population size changes [60]. In order to detect signals of a genetic bottleneck, we used MSVAR version 0.4.1 [56] to estimate the ratio of effective population size change *r*, defined as *N*_*crnt*_ /*N*_*stbl*_, where *N*_*crnt*_ is the current inbreeding effective size and *N*_*stbl*_ is the ancestral stable inbreeding effective size (i.e., before effective population size change). The *r* ratio is expressed in log_10_. Thus, if *r* is negative, the population has declined; if *r* = 0, the population has remained stable; and if *r* is positive, expansion is indicated. MVSVAR version 0.4.1 also estimates θ defined as *2N*_*crnt*_μ, where μ is the mutation rate and *tf* is the time interval of the inbreeding effective population size change in generations scaled by *N*_*crnt*_. The software was run for 2x10^9^ steps recording 20,000 points from the posterior distribution. The upper and lower bounds for the parameters θ, *r*, and *tf* were (-5, 2), (-5.5, 1.5), and (-1, 1), respectively. The limits for *r* comprised both population reduction and population expansion. If 95% of the High Density Interval (HDI) of the posterior distribution did not reach zero, we considered this as a signal of reduction on inbreeding effective population size. To quantify the time in years since the population size started to change (*Tfa*) and the ancestral stable and current inbreeding effective population sizes (hereafter *N*_*stbl*_ and *N*_*crnt*_), we used the software MSVAR version 1.3 [57]. The number of steps and recorded points was the same as for MSVAR version 0.4.1, and the mean and variance for *N*_*stbl*_, *N*_*crnt*_ and *Tfa* were (4, 2.25), (4, 2.25), (5, 2.5). The generation time for each species was considered as the mean time for female sexual maturity; all values were taken from AnAge Database [61]. For both programs, five independent runs were performed with different random seeds; for MSVAR 1.3, the mean and the variance for the initial values were varied for each parameter and for each run as recommended by the authors. To check Monte Carlo Markov Chain (MCMC) convergence, the Gelman-Rubin statistic [62] was used as suggested by the software authors, using the coda package [63] implemented in R version 2.12.1 [64]. The last half of each run was combined to produce a 50,000-step output. The analyses of all posterior distributions were carried out using the packages locfit [65] and hdrcde [66]. In order to avoid false population-collapse signals due to genetic structure [16], we analyzed the TI data considering the clustering results obtained with STRUCTURE. The value of each parameter (*N*_*stbl*_, *N*_*crnt*_ and *Tfa*) was reported as the mode of the posterior distribution, using the first and third quartiles as a measure of distribution dispersion.

To test for differences among populations in the *N*_*crnt*_ and *N*_*stbl*_ estimates, we used the Robust Bayesian Estimate (RBE) [67]. This approach allows a discrete decision about the null value (in this case, that the difference between two estimates is zero). It also provides an estimation of the differences among parameter values, expressed as the mean differences of the marginal posterior distributions including a 95% HDI (High Density Interval). This analysis was performed using the BEST package [67] implemented in R using the default settings.

### TI population Approximate Bayesian Computation analysis and scenario simulations

It has been reported that MSVAR is not suitable for inferring very recent population size changes [60], as in the case of the TI foundation. In order to investigate the extent of this bottleneck, we performed an Approximate Bayesian Computation (ABC) analysis. The aim of the ABC analysis was to approximate the *N*_*crnt*_ of the source population (in this case, the Sonora population) that could explain the actual genetic diversity observed in the TI population (Figure S1). We simulated demographic scenarios considering different bottleneck intensities. The actual date of TI foundation (32 years at the time of sampling) and the actual number of founders [24] were incorporated in the simulations.

The ABC analysis was performed using the DIY-ABC software, version 1.0.4.37 [68]. The scenarios considered were the following: No bottleneck (Scenario A), with Sonora *N*_*crnt*_ fixed at 271; reduced bottleneck (Scenario B), with Sonora *N*_*crnt*_=385; strong bottleneck (Scenario C) using Sonora *N*_*crnt*_=500; and severe bottleneck (Scenario D) at Sonora *N*_*crnt*_=918 (for details, see Figure S1). The parameters for the ABC analysis used were *Tfa*, *N*_*stbl*_ and *N*_*crnt*_ estimates obtained from MSVAR. Additionally, we used the actual time of foundation (approximated at 15 generations ago) as well as the actual number of founders (16 individuals [24]) (Figure S1). The molecular markers simulated for the ABC analysis correspond to 12 autosomal microsatellite loci with a mean mutation rate (previously obtained with MSVAR) of 3.5x10^-4^ and a mean P coefficient of 0.22 (to avoid a strict stepwise mutation model). All loci had a 2 bp motif tandem repeat varying in range from of 20 to 40 bp in length. All summary statistics available were calculated and each scenario had 4 million simulations, from which 0.1% was used for parameter estimation. The scenario selection was carried out using the statistical tests implemented by the software.

In addition, to infer the possible impact on the genetic diversity using TI as a source of individuals for founding new populations, we simulated the possible outcome if new populations were founded from the TI population using 8, 16, 32 and 64 individuals (Figure S1). The simulations considered that founded populations had an instant growth to an *N*_*crnt*_ equal to that estimated for TI. One thousand simulations were performed for each simulated population, and the analyses of the data sets were performed with Genepop using a Perl script (available upon request). The parameters recorded were *H*_*O*_ *, H*_*E*_, and *n*_*a*_. To evaluate the effects on *N*_*crnt*_ on the simulated populations, one data set was selected at random and was analyzed using MSVAR version 1.3 using the settings reported above. All graphics and statistical analysis were performed with R.

### Re-analyses of managed artiodactyl populations from previously published data

In order to compare the *N*_*eI*_-based approach with other populations and species, we re-analyzed seven populations of bighorn sheep (Table 1): four populations from Oregon, USA (HMO, LGO, SMO, JDO; for details see Table 1); one population from Nevada, USA (SRN) (Californian bighorn sheep, *O.c. californiana*); one population from New Mexico, USA (RRNM) (the desert sheep, *O*.*c. mexicana*); and one from Alberta, Canada (SRA) (the mountain sheep, *O*.*c. canadensis*) [29,30]. We also included data from three USA bison (*Bison bison*) populations [69]: one population from Yellowstone National Park (YNP), founded with native members of the locality and from private herds; and one population each from Wind Cave National Park (WCNP) and Sully’s Hill National Game Preserve (SUH), mainly founded from private herds and zoos. We chose these bison populations because they have similar genetic diversity values and allele numbers but different population sizes, thus representing a good opportunity to investigate the relationship among *H*_*E*_, census size, and *N*_*crnt*_ (Table 1). In order to compare species with different habitats and distribution, we included a population from another member of the Bovidae family, the Arabian oryx (*Oryx leucoryx*), from a protected area in Saudi Arabia [70] and from a captive population from China of the black muntjac (*Muntiacus crinifrons*) of the Cervidae [71] (Table 1). The *N*_*crnt*_ estimation for all data sets was carried out with MSVAR using the scheme previously described for the TI population.

**Table 1:** Species, locality, sample size (*N*) and number of loci (*L*), estimated census size (*N*_*census*_), observations regarding each population, and reference.

**Species**	**Locality**	**N/L**	***N_census_***	**Observations**	**Reference**
*Ovis canadensis mexicana*	Tiburon Island, Sonora, México (TI)	63/12	650–700	Sixteen founders from a single source. Continuous population growth.	This work, 24
*Ovis canadensis mexicana*	Red Rock Refuge, New Mexico, USA (RRNM)	25/10	100–200	Captive herd derived from one source, San Andres Mt. Used as a translocation stock.	30
*Ovis canadensis canadensis*	Sheep River, Alberta, Canada (SRA)	55/10	Local population about 60–150	Historically large population, frequent contact with other herds. Past declines due epidemics.	30
*Ovis canadensis californiana*	Hart Mountain, Oregon, USA (HMO)	16/11	270	Twenty founders from a single source (Williams Lake), decline of the population. Isolation since establishment.	29
*Ovis canadensis californiana*	Leslie Gulch, Oregon, USA (LGO)	23/11	125	Seventeen founders from Hart Mt, posterior introduction of 72 individuals from Hart Mt or Steens Mt.	29
*Ovis canadensis californiana*	Steens Mountain, Oregon, USA (SMO)	18/11	185	Multiple releases from Hart Mt (152 in total), decline of the population.	29
*Ovis canadensis californiana*	John Day River, Oregon, USA (JDO)	19/11	310	Multiple introductions, most from Hart Mt (50 in total).	29
*Ovis canadensis californiana*	Santa Rosa Mountains, Nevada, USA (SRN)	31/11	295	Introductions from multiple sources (53 in total).	29
*Bison bison*	Sully’s Hill National Game Preserve, North Dakota, USA (SUH)	29/14*	35	Nineteen founders from five sources. Species under severe bottleneck.	69
*Bison bison*	Wind Cave National Park, South Dakota, USA (WCNP)	345/14*	350	Twenty founders from two sources (14 from a zoo and 6 from YNP).	69
*Bison bison*	Yellowstone National Park, Wyoming, USA (YNP)	505/14*	3,000	Fifty-one founders from three sources (30 indigenous, 21 from two private herds).	69
*Oryx leucoryx*	Mahazat As-Sayd Protected Area, Saudi Arabia (MNWSA)	24/7	200	Twenty-one samples taken from a protected area, founded from seven distinct groups. Three samples taken from a semi-captive population.	70
*Muntiacus crinifrons*	Hefei Wild Animal Park, China (HWCH)	14/11	45	Lower diversity than in the wild. Population founded from a single wild source.	71

* Fourteen loci selected by the authors in the original study due to the large number of alleles.

## Results

### TI population genetic diversity and structure

We found 40 alleles for the 12 microsatellite loci analyzed. The number of alleles ranged from 2 to 7, with an average of 3.33 (S.D. 1.435) (details for each marker can be found in Tables S2 and S3). In general, the number of alleles per locus was lower than previously reported for *O. canadensis* [30,41] but higher than those reported by Hedrick et al. [36] for the same population (see Discussion). The Micro-checker analysis showed the presence of null alleles in locus *OarFCB266*. However, this locus was kept for future analysis because our simulations showed that a genetic bottleneck, like the one that occurred in the TI foundation, could generate an excess of homozygotes that could lead to a false null allele signal (see *TI population ABC analysis* section). The overall values of *H*_*E*_ and *H*_*O*_ were 0.501 (S.D. 0.155) and 0.472 (S.D. 0.159) (Table 2). There were notable differences among loci; for example, *MAF48* showed *H*_*E*_=0.238 with 2 alleles, while *BM848* had *H*_*E*_= 0.738 with 7 alleles (Table S2). Although positive and negative values of *F*_*IS*_ were obtained at different loci (Table S2), only *OarFCB266* (in accordance with the null alleles signal) and *BM1818* had significant values (*F*_*IS*_=0.3629, p=0.011 and *F*_*IS*_=-0.0016, p=0.044, respectively). The overall locus analyses showed a slight, but significant, positive *F*_*IS*_ value of 0.058, indicating a departure from H-W proportions for heterozygote deficit (*p*=0.008). Tests for linkage disequilibrium showed that 7 of 66 tests had a significant value (p < 0.05). It is worth mentioning that the loci used for this study are located on different chromosomes, except for *OarFCB128* and *BM2113*, which are located in chromosome 2 (but have a distance > 250 cM). However, these loci did not show significant linkage values. We interpret this result as a signal of a population bottleneck, as linkage disequilibrium can be associated with population size reduction and genetic drift [72]. The STRUCTURE analyses recorded the highest posterior probability at *k*=1; hence, the TI population was considered as a single population for the MSVAR runs.

**Table 2:** Values of *H*_*E*_, *n*_*a*_, the ratio of population size change (r) expressed in log_10_, current (*N*_*crnt*_), ancestral stable (*N*_*stbl*_) inbreeding effective population size, and time in years to population size change (*Tfa*).

		***H*_*E*_ (S.D.)**	***n*_*a*_ (S.D.)**	***r***	***N*_*crnt*_ (1^st^–3^rd^ quartiles)**	***N*_*stbl*_ (1^st^–3^rd^ quartiles)**	***Tfa* (1^st^–3^rd^ quartiles)**
*O.c. mexicana*	TI	0.501 (0.155)	3.33 (1.435)	-2.192	271 (145–436)	10,522 (6,237–20,941)	3,155 (1,517–6,123)
*O.c. mexicana*	RRNM	0.36 (0.268)	2.4 (0.843)	-2.817	191 (63–289)	12,148 (6,823–26,792)	3,211 (862–5,728)
*O.c. canadensis*	SRA	0.596 (0.153)	4.4 (1.173)	*	388 (158–585)	10,551 (6,339–17,906)	1,857 (723–3,457)
*O.c. californiana*	HMO	0.35 (0.262)	2.22 (1.09)	-2.774	62 (25–101)	56,865 (27,415–123,027)	2,951 (1,196–5,202)
*O.c. californiana*	LGO	0.34 (0.220)	2.33 (0.71)	-3.135	42 (15–72)	43,822 (21,379–86,497)	1,508 (564–2,756)
*O.c. californiana*	SMO	0. 32 (0.254)	2.22 (0.97)	-3.287	37 (12–62)	46,206 (24,099–93,111)	1,404 (500–2,518)
*O.c. californiana*	JDO	0.39 (0.232)	2.44 (0.88)	-2.892	57 (22–99)	39,210 (20,464–74,645)	1,938 (725–3,188)
*O.c. californiana*	SRN	0.57 (0.211)	3.78 (1.39)	-2.504	102 (34–179)	25,194 (14,622–44,055)	1,409 (478–2,477)
*B. bison*	SUH	0.604 (0.137)	4 (1.35)	*	45 (12–83)	17,853 (11,588–29,512)	440 (140–797)
*B. bison*	WCNP	0.650 (0.141)	4.92 (1.859)	-2.240	103 (33–162)	19,006 (11,885–30,690)	732 (264–1,229)
*B. bison*	YNP	0.619 (0.120)	4.17 (1.13)	*	220 (78–350)	24,980 (15,812–40,087)	1,803 (664–3,133)
*O. leucoryx*	MNWSA	0.565 (0.078)	3 (0.816)	-4.210	361 (163–587)	77,821 (24,889–242,103)	11,837 (3,606–24,266)
*M. cinifrons*	HWCH	0.675 (0.137)	5.3 (1.368)	*	487 (195–771)	77,357 (48,641–122,744)	1,508 (610–2,415)

* Different runs did not converge as indicated by the Gelman-Rubin statistic (97.5% quantiles >1.02).

The TI population showed strong signals of a past genetic bottleneck, as the MSVAR *r* estimate was -2.192, while the estimates of the current (*N*_*crnt*_) and ancestral stables (*N*_*stbl*_) were 271 (145–436, 1^st^–3^rd^ quartiles) and 10,522 (6,237–20,941, 1^st^–3^rd^ quartiles) individuals. Nevertheless, the date of the event of population decrease did not correspond to the TI foundation, as the *Tfa* estimate was 3,155 (1,517–6,123, 1^st^–3^rd^ quartiles) years ago (Table 2).

### Mitochondrial genetic diversity

We recovered the two TI haplotypes previously reported for the species in Genbank (Accession numbers: AY116621, AY116622). The haplotype AY116621 was predominant (present in 97% of the sample). There were 11 segregating sites, the haplotype diversity (*H*) was 0.125 (S.D. 0.055), and the nucleotide diversity (*π*) was 0.00249 (S.D. 0.0011). 

### TI population ABC analysis

**Figure 2:**
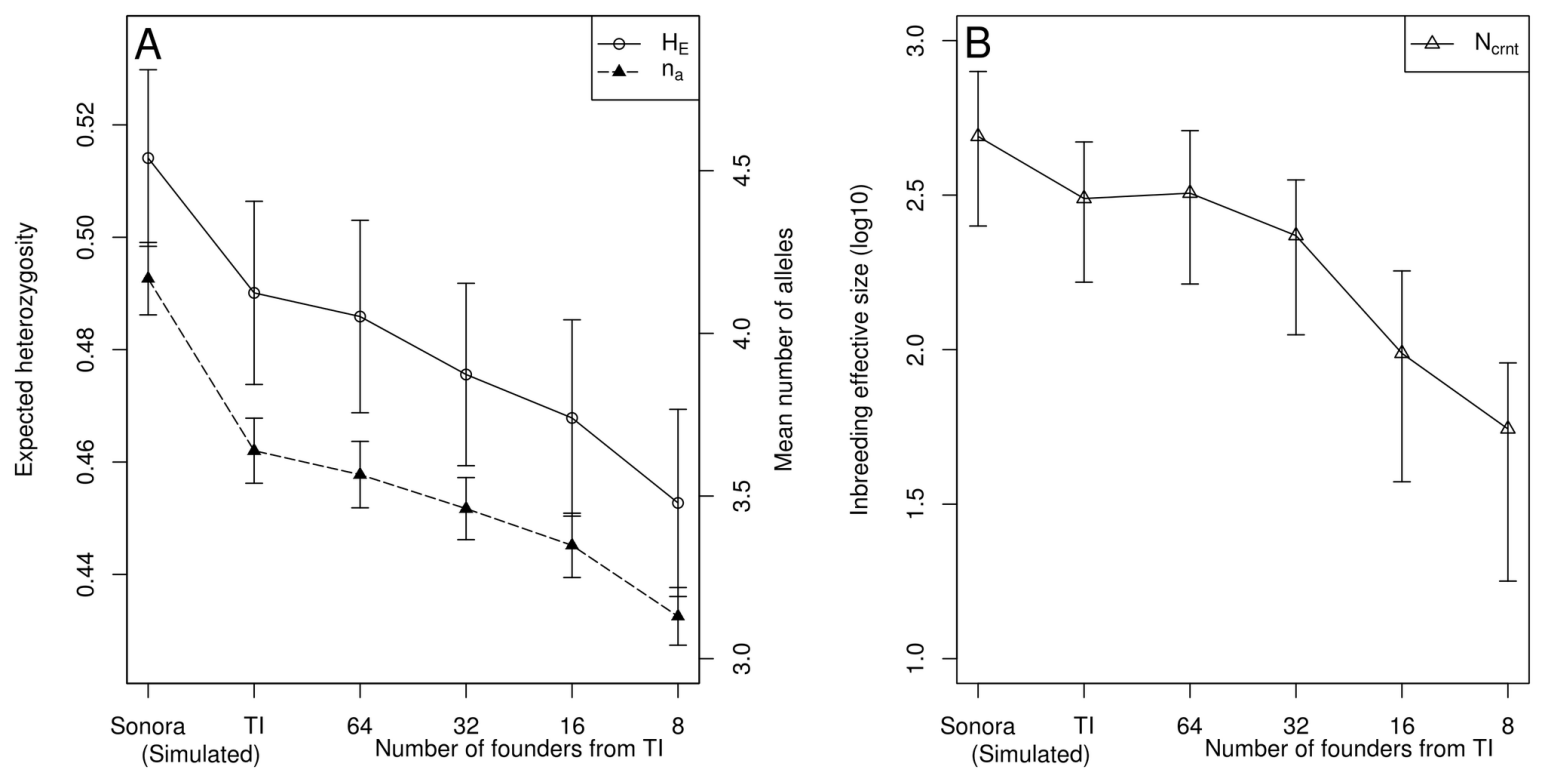
Plots of simulations of the loss of genetic diversity and current inbreeding effective population size. A) Plot of the loss of expected heterozygosity (H_E_) and mean number of alleles (N_a_) for the simulated scenarios. Number of founders correspond to population founded from TI-simulated population. Error bars correspond to standard error using the number of loci as sample size. B) Change in the inbreeding effective size (N_crrnt_) of TI- and Sonora-simulated populations, as well as for the new one founded from TI. Error bars represent first and third quartiles of the parameter distribution.

The ABC analysis showed that Scenario C (Figure S1) had the highest probability, indicating that an *N*_*crnt*_ of 500 individuals for the Sonora source population could explain the genetic diversity observed in the actual TI population. The analysis of the TI-simulated data sets showed an average value of *H*_*E*_=0.4897 (S.D. 0.0139) and *n*_*a*_ =3.639 (S.D. 0.3465), while the Sonora-simulated population had *H*_*E*_ =0.5136 (S.D. 0.0128) and *n*_*a*_ of 4.168 (S.D. 0.317). In accordance with the real data set, we observed the presence of null alleles in a random sample of 100 simulations taken from the TI-simulated data (analyzed with Micro-checker). A g-test performed against a binomial distribution [73], based on 5% of the random variation, showed that the observed results departed from a stochastic process (p< 0.005). This finding suggests that the null allele signal observed in the *OarFCB266* locus in the real TI population could be explained by a deficiency of heterozygotes on this locus. This deficiency could be caused by the population size reduction, due to the TI foundation, more than an artifact of the PCR amplification. As expected, the Sonora-simulated population showed higher diversity (t-test *p*=0.0002) than the TI-simulated population. In the hypothetical populations, we observed a steady decrease in *H*_*E*_ and *n*_*a*_ as the number of founders was reduced (Figure 2A). This tendency can also be observed in the *N*_*crnt*_ estimates (Figure 2B, Tables S4 and S5).

### Current and ancestral stable inbreeding effective sizes and dates of size change of re-analyzed data sets

As shown in Table 2, all populations of bighorn sheep and other species showed signals of population decline. The MSVAR *r* estimate ranged from -2.24 for WCNP to -4.21 for MNWSA. We observed that the Gelman-Rubin statistic showed that some populations did not reach convergence (SRA, HWCH, YNP, and SUH). Nevertheless, as the 95% HDI of the *N*_*crnt*_ and *N*_*stbl*_ posterior distributions did not overlap, we take this result as a significant signal of population size change.

**Figure 3:**
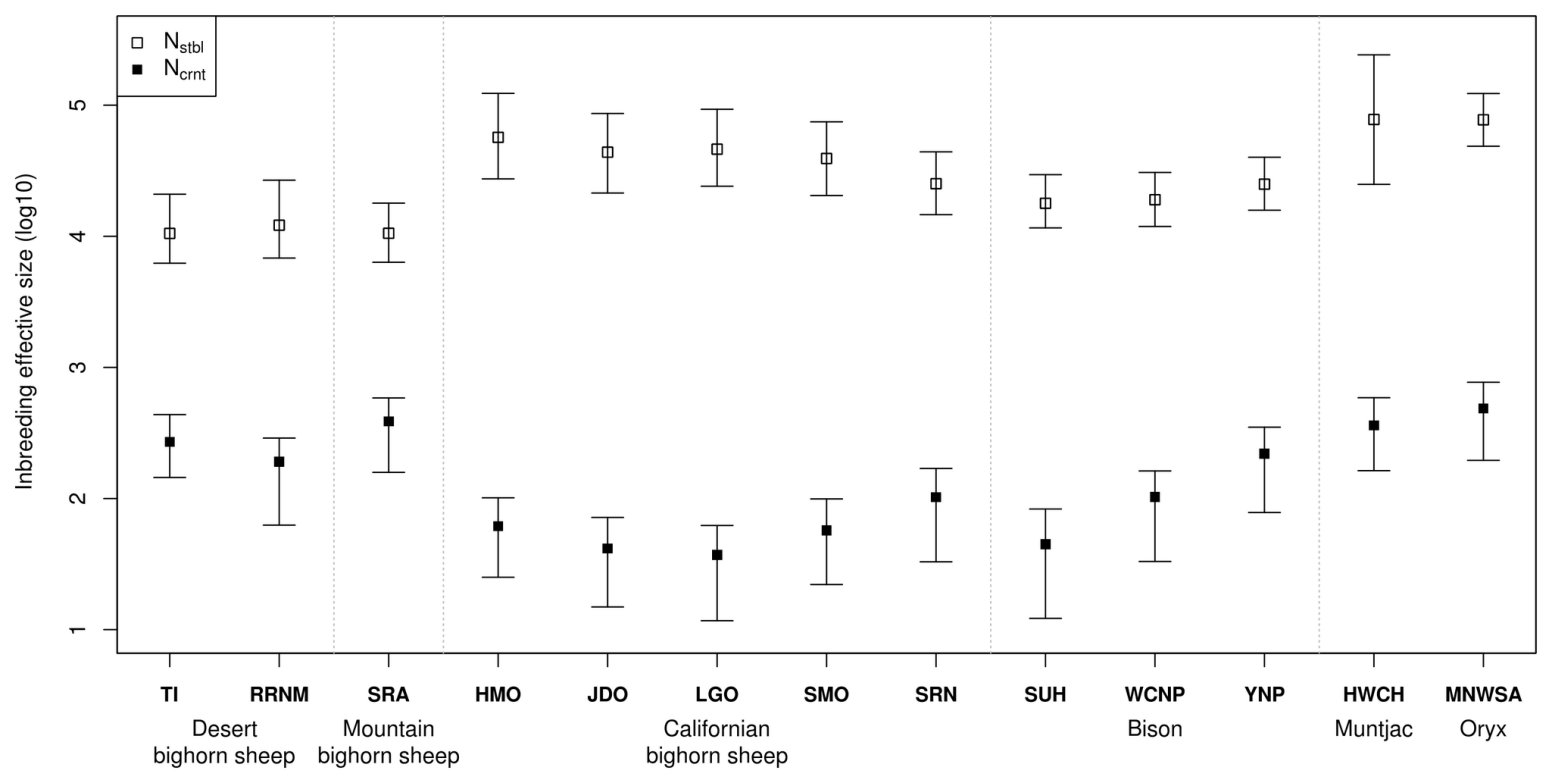
Estimates for the ancestral stable (N_stlb_) and current (N_crnt_) inbreeding effective sizes. Dots correspond to the modal value of parameter distributions obtained with MSVAR 1.3. Error bars represent the first and third quartiles.

The *N*_*crnt*_ estimates exhibited differences among species and contrasting values within bighorn sheep and bison populations (Figure 3, Table 2). The *N*_*crnt*_ obtained for bighorn sheep ranged from 37 (12–62, 1^st^–3^rd^ quartiles) individuals for SMO to 388 (158–585, 1^st^–3^rd^ quartiles) for SRA (Table 2). In the case of bison, the *N*_*crnt*_ values were 45 (12–83, 1^st^–3^rd^ quartiles), 103 (33–162, 1^st^–3^rd^ quartiles), and 220 (78–350, 1^st^–3^rd^ quartiles) for SUH, WCNP, and YNP, respectively. The *N*_*crnt*_ estimates for the black muntjac (HWCH) were 487 (195–771, 1^st^–3^rd^ quartiles) and 361 individuals (163–587, 1^st^–3^rd^ quartiles) for the Arabian oryx (MNWSA). According to the Robust Bayesian Estimate (RBE) analysis, all paired comparisons of the *N*_*crnt*_ estimates between populations showed significant differences (all comparisons are reported in Table S6).

The ancestral stable (*N*_*stbl*_) estimates also had large ranges (Table 2, Figure 3). The *O. canadensis* populations exhibited values from 10,551 (6,339–17,906, 1^st^–3^rd^ quartiles) for SRA to 56,865 (27,415–123,027, 1^st^–3^rd^ quartiles) individuals for HMO. The SUH and WCNP bison populations showed 17,853 (11,588–29,512, 1^st^–3^rd^ quartiles) and 19,006 (11,885–30,690, 1^st^–3^rd^ quartiles) individuals, and the YNP population showed 24,980 (15,812–40,087, 1^st^–3^rd^ quartiles) individuals. The estimates for MNWSA and HWCH were 77,821 (24,889–242,103, 1^st^–3^rd^ quartiles) and 77,357 (48,641–122,744, 1^st^–3^rd^ quartiles). All paired comparisons of *N*_*stbl*_ estimates and their significance are reported in Table S7.

**Figure 4:**
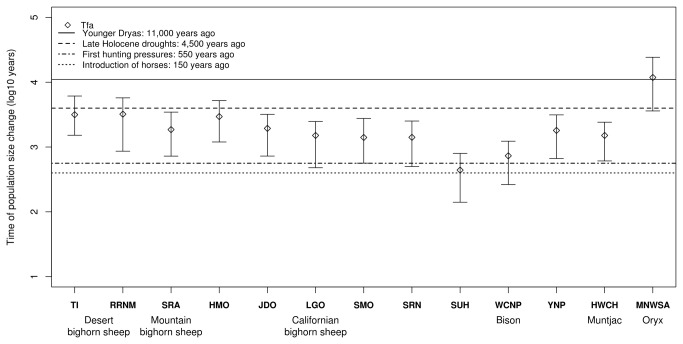
Plot of the time of population size change (Tfa). Solid line corresponds to Younger Dryas (11,000 years ago). The dashed line corresponds to late Holocene droughts (4,200 years ago), the dot-dash line corresponds to the first hunting pressures for bison (550 years ago) and the dotted line corresponds to the introduction of horses to North America (150 years ago). Dots correspond to the modal value of the parameter distribution obtained with MSVAR 1.3, and error bars correspond to first and third quartiles.

The estimates of time of population size change (*Tfa*) are shown in Figure 3 and Table 2. The *O. canadensis* populations ranged from 1,404 years (500–2518, 1^st^–3^rd^ quartiles) for SMO to 3,211 years (862–5,728, 1^st^–3^rd^ quartiles) for RRNM. The bison SUH and WCNP populations showed recent *Tfa* values (440 years, 140–797, 1^st^–3^rd^ quartiles and 732 years, 264–1,229, 1^st^–3^rd^ quartiles), while YNP had 1,803 (664–3,133, 1^st^–3^rd^ quartiles) years. HWCH showed a similar value to bighorn sheep populations (1,508 years, 610–2,415, 1^st^–3^rd^ quartiles) while MNWSA was rather ancient (11,837 years, 3,606–24,266, 1^st^–3^rd^ quartiles). Except for MNWSA, most of the *Tfa* results were similar and their interquartile intervals overlapped. Even more, some populations presented almost the same *Tfa* estimate (e.g., TI and RRNM, Table 2, Figure 4).

## Discussion

### Genetic diversity and management of TI population

The *H*_*E*_ estimated for TI was lower than the reported free-ranging or wild *O. canadensis* populations (e.g., SRA) but was higher than the estimates for other managed populations [29,30]. Our results differ from those of Hedrick et al. [36], as we find higher *H*_*E*_ values (0.501 vs 0.420). However, this difference could be due to the difference in sample size that led to a sub-sampling of alleles and, therefore, a genetic diversity underestimation (we recovered one more allele on two of the same loci analyzed). Nevertheless, our analyses indicate that the TI population is not free from a genetic bottleneck. The simulations showed that TI suffered a bottleneck that reduced its *N*_*crnt*_ to about half of the source population, and that the use of TI as a source for translocation of individuals could lead to a severe *N*_*crnt*_ reduction, especially when a low number of founders are used. For example, using 16 founders the genetic diversity loss for *H*_*E*_ was 3% and for *N*_*crnt*_ was 25%; meanwhile, using 8 founders we observed a reduction of 8% in *H*_*E*_, and *N*_*crnt*_ was reduced by about 82% (Figures 2A and 2B, Table S4). Considering our results, we do not recommend the use of the TI population as the only source for restocking other populations. Instead, we suggest the use of individuals from several sources in order to prevent the reduction of effective size and the consequent diminution of genetic variation [6,9,11,29,30,36].

So, what could explain the apparent success of the TI population and the relatively high levels of genetic diversity? It has been argued that the lack of predators, as well as the suitable habitat, promoted a steady growth of the population [25,74]. However, the demographic growth itself does not ensure the retention of genetic diversity. For example, a demographic increase with a continuous reduction in genetic diversity due to genetic drift and inbreeding was reported in mountain goats (*Oreamnos americanus*) [19]. The authors stated that this could be a consequence of a small *N*_*e*_ caused by the mating system of the species and the isolation of the population [19]. In the case of bighorn sheep, it has been reported that sexual segregation is strongly influenced by the presence of predators [75], and that in their absence, the herds can have a more homogeneous male-female proportion allowing ewes to mate with several males despite the dominance hierarchy [76]. This minimizes the effects on *N*_*e*_ due to an uneven male allelic contribution by polygynous behavior [77,78]. However, no studies have been done on the fine-scale population dynamics of TI; therefore, it is unknown at the present time if different groups of animals may act as isolated herds. In this sense, the estimated *F*_*IS*_ could be due not only to TI foundation, but also to the Wahlund effect caused by the social structure of the herds in TI. Another factor promoting high levels of genetic diversity is natural selection. Kaeuffer et al. [35] observed that in a single-pair founded mouflon (*Ovis aries*) population, genetic diversity could be influenced by natural selection, thus overtaking the effects of genetic drift. In this sense, it has been recognized that natural selection plays an important role in maintaining genetic diversity in bighorn sheep [28].

### Current and ancestral stable inbreeding effective sizes and genetic diversity

In general, we found a positive relationship between *H*_*E*_ and *N*_*crnt*_. However, the results also showed deviations from this pattern (Table 2). For example, for *O. canadensis* populations, the TI population showed an *N*_crnt_ estimate larger than SRN; however, SRN had a higher *H*_*E*_ than that of TI (Table 2, Table S6). Similarly, RRNM had a relatively low *H*_*E*_, but its *N*_*crnt*_ was higher than the *N*_*crnt*_ for all the populations from Oregon (HMO, LGO, SMO, and JDO) (Table 2, Figure 2). The *H*_*E*_ reported for the SRA population was ~5% larger than that reported for SRN (Table 2), while the estimated *N*_*crnt*_ for SRA was about two times larger than that estimated for SRN (Table S6). In the case of bison populations [64] (Table 2), the reported *H*_*E*_ for the three populations were similar, but our *N*_*crnt*_ estimates were contrasting. The *N*_*crnt*_ estimate for YNP is larger than that of WCNP, although WCNP had higher *H*_*E*_ than YNP (Table 2, Table S6). Instead, YNP has relatively high levels of *H*_*E*_ and *N*_*crnt*_. This could be explained by the fact that the YNP population had a large census size and was founded using native animals. In contrast, SUH showed a considerably low *N*_*crnt*_, even though this population had a *H*_*E*_ of 0.604 (Table 2).

The black muntjac (HWCH) and Arabian oryx (MNWSA) populations had the largest *N*_*crnt*_ estimates, yet they have small census sizes, especially the black muntjac. In the case of HWCH, the population was founded from a single wild source [71]; thus, the estimated *N*_*crnt*_ could correspond to the source population and not necessarily to the actual captive population. MNWSA is similar; however, since this population was founded using several sources instead of a single source, MNWSA could offer a better representation of the source or original genetic pool. For both populations (HWCH and MNWSA), it is likely that not enough time has passed to decrease the “effective size signal” by drift or inbreeding depression and, consequently, to erode the genetic diversity [3,10].

The contrasting patterns between the *H*_*E*_ and *N*_*crnt*_ estimates could be attributed to the fact that genetic variation could be restored more rapidly than the *N*_*crnt*_. In this sense, the introduction of alleles can promote an increase of *H*_*E*_ but not necessarily of *N*_*crnt*_. This could be the case for bighorn populations, such as SRN, SUH and WCNP, that showed relatively high levels of *H*_*E*_ but lower *N*_*crnt*_ when compared with other populations. For MNWSA and HWCH, it is clear that the populations could not retain such genetic diversity, so it is imperative to increase the census number of both populations in order to avoid a future reduction of *N*_*crnt*_ and the associated genetic diversity loss. 

The differences observed between *N*_*stbl*_ and *N*_*crnt*_ were of at least one order of magnitude (Figure 3), suggesting a considerable genetic bottleneck for all species. In the case of the bighorn sheep populations, the TI, RRNM, and SRA *N*_*stbl*_ estimates were similar, while the Californian bighorn sheep populations exhibited the largest and most variable estimates of *N*_*stbl*_ (Table 2, Figure 3). The HMO population was founded with individuals from the Williams Lake population in Canada, while LGO and SMO were founded mostly with individuals from HMO [29]. The *N*_*stbl*_ estimates for these populations actually may represent the ancestral stable effective size of the Williams Lake population, and the differences observed may be due to the sub-sampling represented by the LGO and SMO populations. On the other hand, the difference observed in JDO and SRN could be explained by the fact that these populations were founded from several different sources that could represent a mixed ancestral genetic pool. The bison populations showed similar *N*_*stbl*_ values; however, this was expected considering that bison populations could share the same genetic pool due to translocation history. Nevertheless, the *N*_*stbl*_ estimate for each population is relatively small considering the estimates for the reports of the historical size of bison [79]. These results suggest that in the past, bison populations could have remained as relatively small and isolated demes, so local sampling of herds represented the diversity of each deme. Finally, the non-American species had the largest *N*_*stbl*_ observed by far, which could indicate that their populations were very large and that they had large, interconnected populations that resulted in larger *N*_*stbl*_ estimates.

It is necessary to consider the factors that influence and possibly bias our *N*_*stbl*_ estimates: i) the analyzed populations could have historical genetic flow with other populations, and ii) the antiquity of the ancestral stable population size. Historical interconnections with other populations could lead to an *N*_*stbl*_ overestimation, as MSVAR assumes that the sample represents a closed population [56,57]. In addition, Schwartz et al. [80] and Beaumont [16] stated that, on long time-scales, the estimates of *N*_*e*_ obtained from genetic data (in our study, *N*_*stbl*_) could approach the global *N*_*e*_ for the species, yielding historical *N*_*e*_ estimates very different from actual local census size.

### The time of the population size change Tfa, and anthropogenic pressures

The European colonization of the Americas has been considered as one of the most important factors influencing the decline of the bighorn sheep populations [81]. However, our *Tfa* estimates suggested that other factors besides the European settlement could have affected the bighorn sheep populations (Figure 4, Table 2). It has been reported that the climate changes during the Holocene restricted vegetation cover and water availability [82–86]; however, the climate became less arid in the late Holocene, allowing the artiodactyl populations to grow [86]. Nevertheless, archaeological and paleontological records across North America revealed that, although the environmental conditions were favorable for artiodactyls, the hunting pressures by Native Americans appear to have caused substantial declines in artiodactyl populations [86–89]. The *Tfa* values obtained could be related to an ancient and continuous population decrease driven mostly by human foragers who took advantage of the increased high artiodactyl densities [85].

As in the case of the bighorn sheep populations, the Holocene droughts, as well as hunting pressures, could have influenced the bison populations [90]. For example, the *Tfa* for YNP is in accordance with fossil abundance in the locality, having peaked in the late Holocene [90]. However, more recent *Tfa* values were found in SUH and WCNP (Figure 4). It has been documented that considerable anthropogenic pressures on bison populations began around 550 years ago and increased 150 years later with the introduction of horses [91,92]. This finding indicates that the SUH and WCNP populations had a strong population-decline signal, primarily driven by hunting in the last centuries, of such magnitude that it overwhelmed the Holocene influence. In contrast, YNP was the only population that had native individuals; thus, its *Tfa* estimate represents the time of population size change of a relict population, while SUH and WCNP estimates relate to more recent anthropogenic pressures.

Booth et al. [84] reported that severe droughts at ~4.2 kyr are recorded at multiple mid-latitude and subtropical sites on all other continents of the Northern Hemisphere. This suggests that the Late Holocene climate changes could influence artiodactyls in other continents, including black muntjac. Finally, the MNWSA *Tfa* value corresponds to another important climatic event, the Younger Dryas (11,000 years ago) [93]. This event is characterized by a striking increase of aridity. This caused a retreat of herbaceous plants in Southwest Asia [89] that could have influenced oryx populations. The *N*_*eI*_ reduction in this species could reflect a severe influence of climatic changes and/or could be partly due to human activities.

## Conclusions

The estimation of the effective population size provides additional information about genetic diversity. In this sense, our results showed that past human influences and possibly climatic changes played a major role in demographic trends on the artiodactyls studied here. Moreover, the use of *N*_*crnt*_ and *N*_*stlb*_ estimates allows a glimpse of the possible fate of genetic diversity in the future (for example, the effects of genetic drift or posterior bottlenecks). Thus, the effective size estimation, along with classic summary statistics, allows us to make better decisions for planning management and conservation strategies.

## Supporting Information

Figure S1 **Schematic representation of the ABC-analysis scenarios.** From past to present, the simulations are as follows: Begin with an ancestral population that had a constant effective size (N_stlb_); the population had a size change at an exponential rate during the *Tfa* years (in this case, scaled to generations). Fifteen generations before the present, the TI foundation occurred, represented as the continental (Sonora) and TI population split. Finally, in the present (N_crnt_), 63 samples were taken from TI. We considered the actual information on the TI foundation (16 founders, 30 years ago), and parameters *N*_*stlb*_, *N*_*crnt*_ and *Tfa* were obtained from the MSVAR analysis. To determinate the most likely *N*_*crnt*_ of the Sonora source population, four scenarios were considered: (A) no genetic bottleneck fixing size at 271 individuals; (B) reduced considering 385 individuals; (C) strong 500; and (D) severe bottleneck 918. Finally, in order to predict the effects on genetic diversity of using TI as source for founding new populations, we simulated the outcome for 64, 32, 16, and 8 founders using the most likely scenario obtained with the ABC-analysis.
(TIF)

Protocols S1 **Protocols for the amplification and genotyping of the molecular markers used in this study.**
(DOC)

Table S1 **Chromosomal position, primer sequences, MgCl_2_ concentration, alignment temperature, and references of the microsatellite loci used in this study.**
(DOC)

Table S2 **Summary statistics of microsatellite markers used in this study.**
(DOC)

Table S3 **Allele size and frequencies of microsatellite loci used in this study.**
(DOC)

Table S4 ***H*_*E*_ and *n*_*a*_ values obtained from the ABC-analysis.**
(DOC)

Table S5 **Current effective inbreeding population size (*N*_*crnt*_) obtained form the ABC-analysis simulations.**
(DOC)

Table S6 **Paired comparisons of current inbreeding effective population size (*N*_*crnt*_) estimates using Robust Bayesian Estimates (RBE).**
(XLS)

Table S7 **Paired comparisons of ancestral stable effective population size (*N*_*stbl*_) estimates using Robust Bayesian Estimates (RBE).**
(XLS)
