# Supplementary material for: Effective Population Size, Genetic Variation, and Their Relevance for Conservation: The Bighorn Sheep in Tiburon Island and Comparisons with Managed Artiodactyls
Source: PLoS One. 2013 Oct 11;8(10):e78120. doi: 10.1371/journal.pone.0078120 (PMC3795651; doi:10.1371/journal.pone.0078120)
Supplement: Protocols S1 — Protocols for the amplification and genotyping of the molecular markers used in this study. (DOC) [file pone.0078120.s002.doc]

**Protocols S1. Protocols for the amplification and genotyping of the molecular markers used in this study.**

PCR protocol for microsatellite amplification

The PCR reactions had the following protocol: 5 min at 94°C for denaturation, followed by 30 cycles of 30 s at 94°C, annealing for 30 s at the temperature reported on Table S1, and 1 s of extension at 72 °C. All products were visualized on 2% agarose gel stained with ethidium bromide. Fragment analysis were carried out on an ABI 3100 automatic sequencer in 10 µl reaction mixes containing 9.25 µl deionized formamide, 0.25 µl GeneScan^TM^ 600 LIZ® Size Standard (Applied Biosystems) and 0.5-2 µl PCR products, with an initial denaturation step of 95 °C. The software Peak Scanner v.1.0 (Applied Biosystems) were used to estimate the fragments sizes. PCR was carried out in 10 µl reaction volumes containing 25 ng/µl DNA, 1.25 µl 10× buffer, 0.2 mM dNTPs, 0.4 µM forward and reverse primers, and 1 unit of *Taq* DNA polymerase (Applied Biosystems)

PCR protocol for mitochondrial control region amplification.

The PCR conditions for all pair of primers were: 3 min at 94°C for denaturation, followed by 30 cycles of 30 s at 94°C, annealing for 45 s at 54 °C, 2 min s of extension at 72 °C, and a final extension step of 7 min at 72°C. The internal primers were used as follows: Mit_F1/R1_435, F2_144/ R2_529.

Primers used for the mitochondrial control region amplification

|  |  | Reference |
| --- | --- | --- |
| Mit_F1 | Fwd5' AACCTCCCTAAGACTCAA-GG | Boyce *et al*. 1999 |
| R1_435 | Rev5'CRGGATACGCATGTTGACTRGRAC |  |
| F2_144 | Fwd5'AAARCACACCAYCCACCCACGGAC | This work |
| R2_529 | Rev5'ACAATGAATGGGCCCGGAGCGA |  |
