# Supplementary material for: Effective Population Size, Genetic Variation, and Their Relevance for Conservation: The Bighorn Sheep in Tiburon Island and Comparisons with Managed Artiodactyls
Source: PLoS One. 2013 Oct 11;8(10):e78120. doi: 10.1371/journal.pone.0078120 (PMC3795651; doi:10.1371/journal.pone.0078120)
Supplement: Table S1 — Chromosomal position, primer sequences, MgCl2 concentration, alignment temperature, and references of the microsatellite loci used in this study. (DOC) [file pone.0078120.s003.doc]

**Table S1. Chromosomal position, primer sequences, MgCl_2_ concentration, alignment temperature, and references of the microsatellite loci used in this study.**

| Microsatellite locus | Chromosome for *O. aries* | Primer sequence | Conc. MgCl_2_ | Aling.  Temp. | Reference |
| --- | --- | --- | --- | --- | --- |
| *OarFCB266* | 25 | Fwd_5'GGCTTTTCCACTACGAAATGTATCCTCAC  Rev_5'CACCACATACCAAACACACAGCCTGC | 1mM | 58 ºC | Crawford et al., 1994 |
| *OarFCB128* | 2 | Fwd_5'ATTAAAGCATCTTCTCTTTATTTCCTCGC  Rev_5'CAGCTGAGCAACTAAGACATACATGCG | 1mM | 60 ºC | Crawford et al., 1994 |
| *MAF48* | 5 | Fwd_5'TCACTAAACCAGGGGCGG  Rev_5'GAGGCAGGCAAAATCAGAAC | 1mM | 56 ºC | Kappes et al., 1997 |
| *MAF36* | 22 | Fwd_5'CATATACCTGGGAGGAATGCATTACG  Rev_5'TTGCAAAAGTTGGACACAATTGAGC | 1mM | 56 ºC | Crawford et al. 1995 |
| *BM848* | 15 | Fwd_5'TGGTTGGAAGGAAAACTTGG  Rev_5'CCCTCTGCTCCTCAAGACAC | 3mM | 55 ºC | Crawford et al., 1995 |
| BM1818 | 20 | Fwd_5'AGCTGGGAATATAACCAAAGG  Rev_5'AGTGCTTTCAAGGTCCATGC | 3mM | 55 ºC | Bishop et al., 1994 |
| *MAF209* | 17 | Fwd_5'TCATGCACITAAGTATGTAGGATGCTG  Rev_5'GATCACAAAAAGTTGGATACAACCGTGG | 1mM | 56 ºC | Buchanan and Crawford, 1992 |
| D16S3 | 12 | Fwd_5'GCTTTCAGAAATAGTTTGCATTCA  Rev_5'ATCTTCACATGATATTACAGCAGA | 2mM | 58 ºC | Crawford et al., 1995 |
| *BM2113* | 2 | Fwd_5'GCTGCCTTCTACCAAATACCC  Rev_5'CTTCCTGAGAGAAGCAACACC | 1mM | 56 ºC | Crawford et al., 1995 |
| *D12S4* | 12 | Fwd_5'CCTTTCAAAAACACGGAAATTCGGGGG  Rev_5'CTTCAGGCATACCCTACACCACATG | 3mM | 56 ºC | Kappes et al., 1997 |
| *AGLA293* | 3 | Fwd_5'GAAACTCAACCCAAGACAACTCAAG  Rev_5'ATGACTTTATTCTCCACCTAGCAGA | 1mM | 56 ºC | Crawford et al., 1995 |
| MGTG4B | 4* | Fwd_5'GAGCAGCTTCTTTCTTTCTCATCTT  Rev_5'GCTCTTGGAAGCTTATTGTATAAAG | 2mM | 56 ºC | Steffen and Eggen, 1993 |

* Chromosome location reported for *Bos taurus*.

**Table S1 references**

Bishop MD, Kappes SM, Keele JW, Stone RT, Sunden SL, et al. (1994) A genetic linkage map for cattle. Genetics 136: 619-639.

Buchanan FC, Crawford AM (1992) Ovine dinucleotide repeat polymorphism at the MAF209 locus. Anim Genet 23: 83.

Crawford AM, Montgomery GW, Pierson CA, Brown T, Dodds, K.G., Sunden, et al. (1994) Sheep linkage mapping: nineteen linkage groups derived from the analysis of paternal half-sib families. Genetics 137: 573-579.

Kappes SM, Keele JW, Stone RT, McGraw RA, Sonstegard TS, et al. (1997) A second-generation linkage map of the bovine genome. Genome Res 7: 235-249.

Steffen P, Eggen A, Dietz AB, Womack JE, Stranzinger G, et al. (1993) Isolation and mapping of polymorphic microsatellites in cattle. Anim Genet 24: 121-124.
