# Supplementary material for: Effective Population Size, Genetic Variation, and Their Relevance for Conservation: The Bighorn Sheep in Tiburon Island and Comparisons with Managed Artiodactyls
Source: PLoS One. 2013 Oct 11;8(10):e78120. doi: 10.1371/journal.pone.0078120 (PMC3795651; doi:10.1371/journal.pone.0078120)
Supplement: Table S2 — Summary statistics of microsatellite markers used in this study. (DOC) [file pone.0078120.s004.doc]

**Table S2. Summary statistics of microsatellite markers used in this study.**

| Locus |  | n_a_ | H_O_ | H_E_ | F_IS_ |  |
| --- | --- | --- | --- | --- | --- | --- |
|  |  |  |  |  |  |  |
| OarFCB266 |  | 3 | 0.150 | 0.247 | 0.395** |  |
| OarFCB128 |  | 2 | 0.396 | 0.501 | 0.210 |  |
| MAF48 |  | 2 | 0.274 | 0.238 | −0.150 |  |
| MAF36 |  | 3 | 0.306 | 0.339 | 0.098 |  |
| BM848 |  | 7 | 0.677 | 0.738 | 0.083 |  |
| BM1818 |  | 4 | 0.633 | 0.619 | −0.023** |  |
| MAF209 |  | 5 | 0.573 | 0.591 | 0.0298 |  |
| D16S3 |  | 2 | 0.492 | 0.479 | −0.0273 |  |
| BM2113 |  | 3 | 0.508 | 0.479 | −0.0598 |  |
| D12S4 |  | 3 | 0.583 | 0.623 | 0.0643 |  |
| AGLA293 |  | 3 | 0.491 | 0.585 | 0.1608 |  |
| MGTG4B |  | 3 | 0.580 | 0.576 | −0.0073 |  |
|  |  |  |  |  |  |  |
|  | Mean | 3.333 | 0.472 | 0.501 |  |  |
|  | s.d. | 1.435 | 0.159 | 0.155 |  |  |

**significant value, p<0.05
