# Supplementary material for: Effective Population Size, Genetic Variation, and Their Relevance for Conservation: The Bighorn Sheep in Tiburon Island and Comparisons with Managed Artiodactyls
Source: PLoS One. 2013 Oct 11;8(10):e78120. doi: 10.1371/journal.pone.0078120 (PMC3795651; doi:10.1371/journal.pone.0078120)
Supplement: Table S3 — Allele size and frequencies of microsatellite loci used in this study. (DOC) [file pone.0078120.s005.doc]

**Table S3. Allele size and frequencies of microsatellite loci used in this study.**

| *Locus* | *Allele size* | *Frequency* | | *Locus* | *Allele size* | *Frequency* | |
| --- | --- | --- | --- | --- | --- | --- | --- |
|  |  |  |  |  |  |  |  |
| **OarFCB266** | 87 | 0.137 |  | **MAF209** | 107 | 0.429 |  |
|  | 97 | 0.008 |  |  | 109 | 0.071 |  |
|  | 99 | 0.855 |  |  | 111 | 0.024 |  |
|  |  |  |  |  | 113 | 0.468 |  |
| **OarFCB128** | 108 | 0.516 |  |  | 115 | 0.008 |  |
|  | 110 | 0.484 |  |  |  |  |  |
|  |  |  |  | **D16S3** | 134 | 0.388 |  |
| **MAF48** | 118 | 0.148 |  |  | 138 | 0.612 |  |
|  | 122 | 0.852 |  |  |  |  |  |
|  |  |  |  | **BM2113** | 142 | 0.621 |  |
| **MAF36** | 85 | 0.812 |  |  | 146 | 0.371 |  |
|  | 99 | 0.141 |  |  | 148 | 0.008 |  |
|  | 101 | 0.047 |  |  |  |  |  |
|  |  |  |  | **D12S4** | 153 | 0.151 |  |
| **BM848** | 221 | 0.069 |  |  | 155 | 0.476 |  |
|  | 229 | 0.038 |  |  | 157 | 0.373 |  |
|  | 231 | 0.331 |  |  |  |  |  |
|  | 233 | 0.154 |  | **AGLA293** | 198 | 0.302 |  |
|  | 235 | 0.008 |  |  | 200 | 0.556 |  |
|  | 237 | 0.038 |  |  | 202 | 0.143 |  |
|  | 239 | 0.361 |  |  |  |  |  |
|  |  |  |  | **MGTG4B** | 114 | 0.328 |  |
| **BM1818** | 253 | 0.217 |  |  | 120 | 0.117 |  |
|  | 255 | 0.517 |  |  | 124 | 0.555 |  |
|  | 257 | 0.242 |  |  |  |  |  |
|  | 259 | 0.025 |  |  |  |  |  |
