# Supplementary material for: Effective Population Size, Genetic Variation, and Their Relevance for Conservation: The Bighorn Sheep in Tiburon Island and Comparisons with Managed Artiodactyls
Source: PLoS One. 2013 Oct 11;8(10):e78120. doi: 10.1371/journal.pone.0078120 (PMC3795651; doi:10.1371/journal.pone.0078120)
Supplement: Table S4 — HE and na values obtained from the ABC-analysis. (DOC) [file pone.0078120.s006.doc]

**Table S4. *H_E_* and *n_a_* values obtained from the ABC-analysis.** Values in parenthesis, corresponds to standard deviation.

| Simulated population | *H_E_* |  |  | *n****_a_*** |  |
| --- | --- | --- | --- | --- | --- |
|  |  |  |  |  |  |
| **SON** | 0.5141 | (0.05343) |  | 4.16817 | (0.27398) |
| **TI** | 0.4901 | (0.05542) |  | 3.63933 | (0.18967) |
| **64 Founders** | 0.48589 | (0.05822) |  | 3.56583 | (0.14500) |
| **32 Founders** | 0.47558 | (0.05518) |  | 3.46133 | (0.10159) |
| **16 Founders** | 0.46784 | (0.05940) |  | 3.34842 | (0.12271) |
| **8 Founders** | 0.45272 | (0.05663) |  | 3.13008 | (0.11870) |
