# Supplementary material for: Effective Population Size, Genetic Variation, and Their Relevance for Conservation: The Bighorn Sheep in Tiburon Island and Comparisons with Managed Artiodactyls
Source: PLoS One. 2013 Oct 11;8(10):e78120. doi: 10.1371/journal.pone.0078120 (PMC3795651; doi:10.1371/journal.pone.0078120)
Supplement: Table S5 — Current effective inbreeding population size (Ncrnt) obtained form the ABC-analysis simulations. (DOC) [file pone.0078120.s007.doc]

**Table S5. Current effective inbreeding population size (*N_crnt_*) obtained form the ABC-analysis simulations.**

| Simulated population | *N_crnt_* | 1^st^ quartile | 3^rd^ quartile |
| --- | --- | --- | --- |
|  |  |  |  |
| **SON** | 489.779 | 794.328 | 251.189 |
| **TI** | 308.268 | 469.934 | 165.299 |
| **64 Founders** | 320.824 | 511.211 | 163.080 |
| **32 Founders** | 233.871 | 354.160 | 111.686 |
| **16 Founders** | 97.2664 | 179.763 | 37.3680 |
| **8 Founders** | 55.3951 | 90.5733 | 17.8238 |
